# Global Revisit Interval Analysis of Landsat-8 -9 and Sentinel-2A -2B Data for Terrestrial Monitoring

**DOI:** 10.3390/s20226631

**Published:** 2020-11-19

**Authors:** Jian Li, Baozhang Chen

**Affiliations:** 1 School of Remote Sensing and Geomatics Engineering, Nanjing University of Information Science & Technology, Nanjing 210044, China; baozhang.chen@nuist.edu.cn; 2 State Key Laboratory of Resources and Environmental Information System, Institute of Geographical Sciences and Natural Resources Research, Chinese Academy of Sciences, Beijing 100101, China

**Keywords:** data availability, orbit swath, cloud contamination, sensor combination

## Abstract

The combination of Landsat-8, Landsat-9, Sentinel-2A and Sentinel-2B data provides a new perspective in remote sensing application for terrestrial monitoring. Jointly, these four sensors together offer global 10–30-m multi-spectral data coverage at a higher temporal revisit frequency. In this study, combinations of four sensors were used to examine the revisit interval by modelled orbit swath information. To investigate different factors that could influence data availability, an analysis was carried out for one year based on daytime surface observations of Landsat-8 and Sentinel-2A -2B. We found that (i) the global median average of revisit intervals for the combination of four sensors was 2.3 days; (ii) the global mean average number of surface observations was 141.4 for the combination of Landsat-8 and Sentinel-2A -2B; (iii) the global mean average cloud-weighted number of observations for the three sensors combined was 81.9. Three different locations were selected to compare with the cloud-weighted number of observations, and the results show an appropriate accuracy. The utility of combining four sensors together and the implication for terrestrial monitoring are discussed.

## 1. Introduction

Satellite combinations of the polar-orbiting Landsat-8 (launched 2013) and Landsat-9 (proposed for launch in middle 2021) by NASA [1] as well as Sentinel-2A (launched 2015) and Sentinel-2B (launched 2017) [2] by European Space Agency (ESA) offer 10–30-m resolution multi-spectral global land coverage. This will substantially increase moderate-resolution satellite observations available for terrestrial monitoring [3]. The data availability of satellite observations is of great importance to the surface land monitoring capabilities, as more data enable more reliable land cover classification and change detection.

The data availability of satellite surface observations changes spatially and temporally and is complicated due to the fact that different factors influence data availability. Combinations of sensors, taking advantage of the different sensor acquisition patterns, could enable more observations to be collated, thus reducing the temporal revisit interval between consecutive observations. Recently, Li and Roy [4] proved that the combination between Landsat-8 and Sentinel-2A -2B could provide more observations and derive a global median average revisit interval of 2.9 days. However, to date the global revisit interval between the combination of Landsat-8 -9 and Sentinel-2A -2B four sensors together has not been investigated.

Satellite orbit swath geometry, i.e., the spatial overlap of lateral orbit swaths increases with higher latitudes [5], which enables more observations at higher latitude. Solar geometry, i.e., latitudinal and temporal variations in the highest latitude toward the North or South Pole that satellites can observe, is related to the temporal progression of the solar position over a year [6]. Acquisition strategy and operational constraints, i.e., data acquisition strategy, payload, station acquired ability, and instrument issues [7,8] also influence data availability. It is important to note that Landsat-8 does not acquire observations globally, i.e., Landsat-8 observations cover all sun-lit landmasses and near-shore coastal regions having a solar elevation that is greater than 5°. Different imaging priorities are set according to latitude and location, i.e., Landsat-8 scenes in USA are set as a high priority, and no scenes are rejected for growing season monitoring of the Northern Hemisphere [8]. Cloud obscuration, when clouds preclude observations, is significant and complicated, as the global variability of clouds in space and time is considerable during Landsat overpasses [9,10].

In this study, revisit intervals between the combinations of Landsat-8, Landsat-9, Sentinel-2A and Sentinel-2B four sensors were investigated by modeled orbit swath information from the Committee on Earth Observation Satellite (CEOS) Visualization Environment (COVE) tool [11] for 2016. A global land grid, defined by an equal area sinusoidal projection, comprising 7201 × 3601 points, and equivalent to that of [4], was used. The global total number of observations and the cloud-weighted number of observations for the combination of Landsat-8 and Sentinel-2A -2B obtained from the United States Geological Survey (USGS) metadata bulk down service [12] were quantified. Surface reflectance observations for the three different locations were selected to evaluate the accuracy of the cloud-weighted number of observations.

## 2. Data

### 2.1. Orbit Swath Information

Both Landsat-8 and Landsat-9 orbits were sun-synchronous at an altitude of 705 km and 98.22° inclination, with a 15° scanning angle and a 185-km swath width [13,14,15]. The two sensors phased 8 d away from each other, yielding a 16-d repeat cycle, which was reduced to 8 d, when combined. The orbits of Sentinel-2A and Sentinel-2B were at an altitude of 786 km and 98.62° inclination, with a 20.6° scanning angle and a 290-km swath width, providing a 10-d repeat cycle for each sensor or a combined 5-d repeat cycle [16].

The orbit swath coordinate and overpass times for Landsat-8, Landsat-9, Sentinel-2A and Sentinel-2B were modeled using the Committee on Earth Observation Satellite (CEOS) Visualization Environment (COVE) tool. Data acquired in 2016 from 1 January to 31 December were selected for Landsat-8, Sentinel-2A and Sentinel-2B, as in [4]. Landsat-9 modelled orbit swath data were stimulated by Landsat-7 using COVE tool for the reason that Landsat-9 will be placed into the current Landsat-7 orbit with an altitude, inclination and an equatorial crossing time the same as Landsat-8, but phased 8 days away [14,15,17]. The orbit swath was cut into 1-min granules and only daytime granules were used in this study.

### 2.2. Daytime Surface Observation Metadata Records for Landsat-8 and Sentinel-2A -2B

Landsat-8 daytime surface observation metadata records were obtained from the USGS Landsat archive metadata database [12]. The database is defined in the Landsat Collection 1 format, with path and row defined in a 185 km × 180 km Worldwide Reference System (WRS-2), and corner coordinates of each scene in the Universal Transverse Mercator (UTM) projection, referenced to the World Geodetic System 1984 datum [18]. The metadata records briefly describe the Collection 1 products. The cloud-cover fractions for Landsat-8 were defined using the CFMask algorithm [19], which gives the total cloud-cover percentage for each scene. This percentage is stored to two decimal places for Landsat-8 records.

Metadata records for Sentinel-2A -2B were downloaded from the USGS Earth explorer [20]. The Sentinel-2 tiles are first provided in Standard Archive Format for Europe (SAFE) files [7] cutting along orbit swath data, and are defined by splitting each SAFE file into fixed 109 × 109 km projected in the Universal Transverse Mercator (UTM) map projection [2,21]. Each metadata record for Sentinel-2 is represented for each tile.

For all three sensors, observations acquired in 2018 from 1 January to 31 December were selected in this study. Only daytime-acquired imageries and global land acquisitions, which even included Antarctica, were used for Landsat-8. Landsat-8 now has the capability for mapping and monitoring snow/ice and water [22,23], with improved radiometric resolution and geolocation accuracy [24,25,26]. Sentinel-2 observations were filtered by descending orbit.

### 2.3. Surface Reflectance Observations for Landsat-8

Landsat 8 Collection 1 atmospherically corrected the surface reflectance image covering three pixel locations: northwest of Algeria, Sahara desert (30.0° N, 0.0°), northwest of Brazil, Amazon forest (3.138° S, 62.180° W), and South of Sweden (56.842° N, 15.057° E) in the year 2018 were downloaded from the USGS Earth Explorer [20]. These three locations were selected as they have the different land cover types, different latitude/longitude and different cloud conditions. Landsat-8 Operational Land Imager (OLI) Collection 1 Surface Reflectances are generated from the Top of Atmosphere Reflectance, using the Land Surface Reflectance Code (LaSRC) [27], which produces the surface reflectance bands and pixel quality assessment band.

## 3. Material and Methods

### 3.1. Global Average Revisit Intervals for Combination of Landsat-8/9 and Sentinel-2A/2B

The global average revisit interval map was derived on a global land point grid using a sinusoidal equal area projection to provide spatially unbiased sampling [28]. The grid comprised 7201 × 3601 points with a spacing of 0.05°. This map captured the overlap of along-track and across-track swath data from Landsat, as well as orbital shifts of the sensor geometry [29,30].

To derive the average revisit interval for each land grid point, each acquisition from the four sensors was independently tested to determine whether it encompassed the land grid point. This was fulfilled by comparing the corner coordinates of an acquisition with those of the land grid point [31]. Considering the large data volume of the sensors, a pre-sorting algorithm was implemented to filter acquisitions with the central coordinates away from the land grid point by a threshold. After establishing whether the acquisition overpassed the land grid point for all four sensors, they were sorted and merged into a single acquisition queue by order of acquisition time, given they were derived from different sensors. The revisit interval dataset was determined by calculating the time difference between every two consecutive observations. The average value of the revisit interval dataset was assigned to the land grid point. A fill value was given, if there were no acquisitions. After looping through all the land grid points, a global average revisit interval map was established. Because the operation on each land grid point was independent, a multi-thread technology was used to speed up the processing of assessing the grid points. All programs were written in C language.

### 3.2. Global Number of Observation Maps for the Daytime Surface Observations of Landsat-8 and Sentinel-2A -2B

The global land grid points defined in sinusoidal projection were used to derive the daytime surface observations of Landsat-8 and Sentinel-2A -2B. The first step was to establish the acquisition dataset for the three sensors that overpassed each land grid point. The total number of observations were added by counting these datasets. The spacing of land grid points was set to be small enough to capture overlap between satellite observations along-track and across-track. In the along-track direction, the southern part of the overlapping area was discarded, while the northern part was retained. In the across-track direction, the overlapping areas were counted twice, as they represent different observations sensed on different dates. The average cloud cover percentage at each land grid point was derived by averaging the cloud cover percentage of each acquisition in the sensed dataset list for each land grid point. A unique fill value was given if no observations were made at a given grid point.

The cloud-obscured images clearly decrease the number of available images. In an image scene, it was assumed that all the image pixels had the same probability of being cloudy, with a value equal to the percent cloud cover in the image scene. This ensured that the number of cloud-contaminated pixels, i.e., the number of datasets lost, was proportional to the cloud cover percentage. Likewise, all useful pixel observations, i.e., those representing the clear part of the image, were proportional to the fraction of cloud cover subtracted from one. Consequently, the cloud-weighted number of observations accumulated within a given period was obtained by the probability of observations that overpassed the land grid point being clear.

Three pixel locations were selected to evaluate the accuracy of the cloud-weighted number of observations. This was fulfilled by counting the number of clear views for each of the locations through the year 2018. Pixel observations were considered as a clear view only if they were not labelled as median confidence or high confidence cloud in the pixel quality assessment band [32]. The accuracy of the cloud-weighted number of observations was compared with the cloud-weighted number of observations with the number of clear views.

## 4. Results

### 4.1. Global Average Revisit Intervals for Combination of Landsat-8/9 and Sentinel-2A/2B

**Figure 1:**
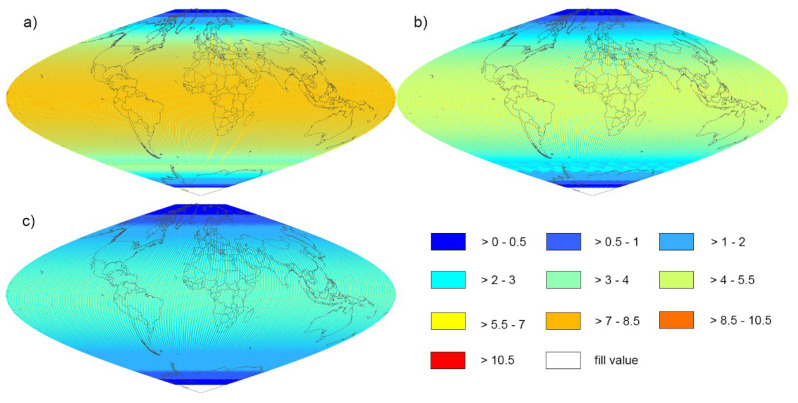
Global average revisit interval (days) maps for the combinations of (**a**) Landsat-8 and Landsat-9, (**b**) Sentinel-2A and Sentinel-2B, and (**c**) Landsat-8, Landsat-9, Sentinel-2A and Sentinel-2B from 1 January to 31 December 2016. The results were examined on a global grid, defined by an equal area sinusoidal projection, composed of 7201 × 3601 grid points, with a spacing of 0.05°. Country boundaries using a sinusoidal projection were overlapped on the maps.

**Figure 2:**
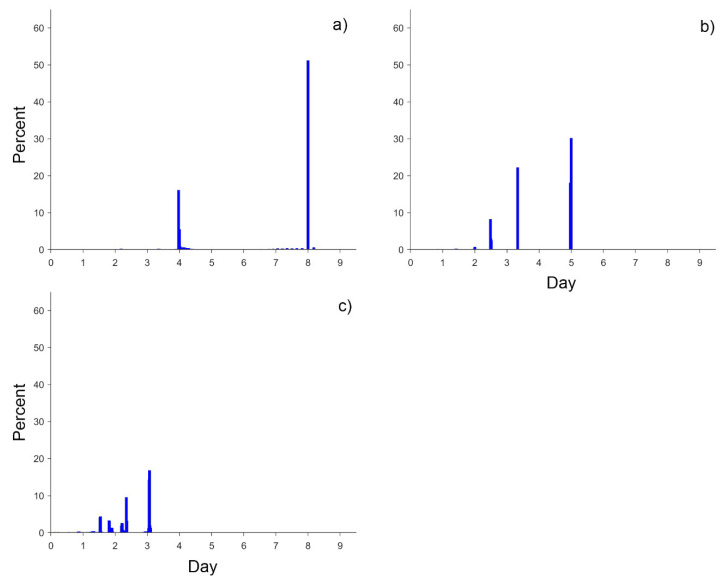
Global average revisit interval histograms divided into 20-min bins for combinations of (**a**) Landsat-8 and Landsat-9, (**b**) Sentinel-2A and Sentinel-2B, and (**c**) Landsat-8, Landsat-9, Sentinel-2A and Sentinel-2B from 1 January to 31 December 2016. The percentages denote the percent of each bin compared with the total number of revisits. All data are from Figure 1.

Figure 1 shows the average revisit intervals derived for each global land grid point for Landsat-8 and Landsat-9 and four sensors combined. The average revisit intervals for Sentinel-2A and Sentinel-2B, reported by [4], are shown for comparison. Given the wider swath width of Sentinel-2, but its longer repeat cycle compared with Landsat, the combinations of Sentinel-2A/2B had a shorter average revisit interval than Landsats-8/9 (Figure 1a,b). As shown in Figure 2, the global revisit interval histograms are not normally distributed because of the variable overlap of the orbits of different sensors and convergence of their orbits at high latitudes. The values beyond 9.0 days were not shown in Figure 2 for the low appearance (account for 0.028%, 0.007% and 0.000% of total grid points, respectively). Table 1 summarises the global mean, median, first mode and the second mode revisit interval data for the various sensor combinations explored in this study.

The combination of more sensors and the utility of their orbit swaths facilitated more observations at given land grid point and decreased the revisit interval between consecutive observations, as seen in Figure 1a–c. The global median average revisit intervals were: 8 d for Landsat-8/9, and 3.7 d for Sentinel-2A/2B. When four sensors were combined, the utility of their different swaths decreased the median average revisit interval to about 2.3 d.

### 4.2. Global Number of Observations for Landsat-8 and Sentinel-2A -2B

**Figure 3:**
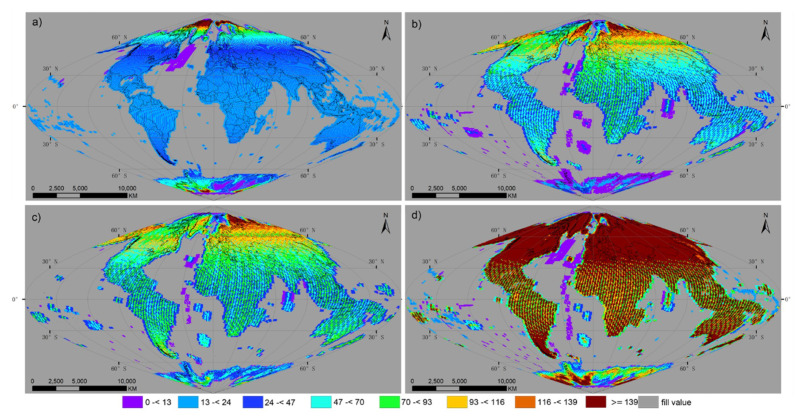
Average number of satellite observations over each land grid point from 1 January to 31 December 2018 by (**a**) Landsat-8, (**b**) Sentinel-2A, (**c**) Sentinel-2B, and (**d**) three sensors combined. The results were examined on a global grid, defined by an equal area sinusoidal projection, composed of 7201 × 3601 grid points, with a spacing of 0.05°.

Figure 3 shows the number of surface land observations for each of the land grid points for Landsat-8, Sentinel-2A, Sentinel-2B and three sensors combined in 2018. Where there were no observations, the land grid point was colored grey. To make the global map spatially explicit, country boundaries as well as latitude and longitude grids were overlapped, using a sinusoidal projection interval every 30°.

The total number of surface observations of Landsat-8 derived for 2018 show a complex pattern (Figure 3a). Clearly, different data reception strategies and orbit geometry influenced the data availability. Most of the Landsat-8 observations were located on land, but there were several observations over oceans. This is because Landsat-8 carried out limited night imaging to monitor active volcanoes and islands worldwide, as both these targets were set to have high imaging priority [8]. Landsat-8 acquired more images at high latitudes, especially above a latitude of 60°, because its swaths overlap more at high latitudes. Combining more sensors enables more data observations, which can be seen from Figure 3a–d. In terms of the number of observations per land grid point during 2018 for Landsat-8, Sentinel-2A, Sentinel-2B and three sensors combined, the global mean averages were 35.67, 57.97, 62.66 and 141.40, respectively.

**Figure 4:**
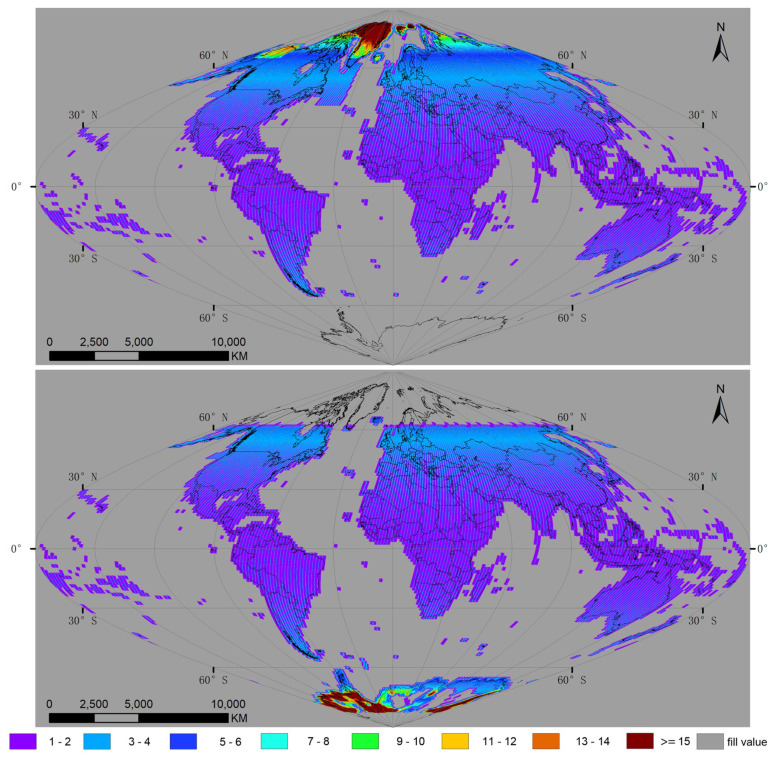
Total number of Landsat-8 observations sensed over each land grid in June 2018 (upper panel) and December 2018 (lower panel). The results were examined on a global grid, defined by an equal area sinusoidal projection, composed of 7201 × 3601 grid points, with a spacing of 0.05°.

Figure 4 shows the average number of Landsat-8 satellite observations over each land grid point for June (upper panel) and December (lower panel) of 2018. These two months were selected because, during the summer solstice (21 June) and winter solstice (22 December), the North Pole has its maximum and minimum tilt towards the Sun, respectively. Considering the repeat cycle of Landsat-8 is 16 d, there should be no more than two observations in any 1-mo period, but the observed number of observations may be greater than two because of the overlap of lateral swaths and the convergence of its orbit at a higher latitude. In fact, the average global number of acquisitions for Landsat-8 was 3.17 in June and 3.05 in December, respectively.

Given the annual progression of the solar position, the geographic coverage of the polar area varies, as the satellite track moves into darkness [30]. The maximum geographic coverage of Landsat-8 towards the south is 55.08° S in June, while there are no observations above a latitude of 66.71° N by Landsat-8 in December.

**Figure 5:**
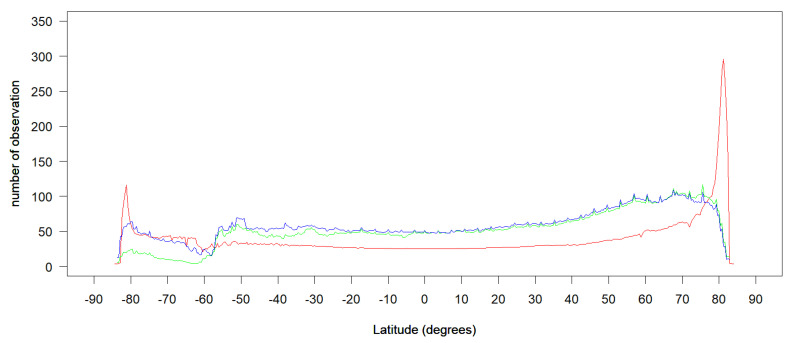
Latitudal mean average number of observations from -90° S to 90° N, for Landsat-8 (red), Sentinel-2A (green), Sentinel-2B (blue) in 2018. Each point was derived by averaging all values from the map of the global total number of observations (Figure 3) along a given latitude, discarding fill values.

Satellite orbit sensor geometry clearly influences global data availability, because the lateral swath convergence at higher latitudes produces more observations at a given grid point. Figure 5 shows the mean average total number of observations by averaging all values along a given latitude (Figure 3), except for fill values for Landsat-8 (red), Sentinel-2A (green), and Sentienl-2B (blue). This map shows the change in the number of observations from the South Pole to the North Pole for three sensors in 2018.

Generally, the latitudinal mean average number of observations for Sentinel-2 is higher than Landsat-8 between the 60° N and 60° S due to Sentinel-2′s wider orbit swath and shorter revisit interval. Landsat-8 acquires more satellite observations on the two pole area for the image acquiring strategy [8]. The latitudinal mean average number of observations had lowest values at 0° latitude in the equatorial region (25.44 for Landsat-8, 50.32 for Sentinel-2A, 51.54 for Sentinel-2B), but increased to maximum values at a latitude of 81.2° N (295.67) for Landsat-8, 75.6° N (116.97) for Sentinel-2A and 67.6° N (109.20) for Sentinel-2B in the Northern Hemisphere and 81.2° S (116.55) for Landsat-8, 50.8° S (60.12) for Sentinel-2A and 50.8° S (69.07) for Sentinel-2B in the Southern Hemisphere. A trough in observation number occurred around 60° S for all three sensors, because at this latitude most of the earth is occupied by ocean, with few land observations (Figure 3). Beyond 81.2° N in the Northern Hemisphere and 81.2° S in the Southern Hemisphere, the mean average latitudinal value deceased sharply for Landsat-8, yielding low values at latitudes of 84.0° N (4.00) and 84.4° S (3.68), where Landsat-8 reached the limit of its geographic coverage and few daytime observations were acquired. Above 84.0° N and 84.4° S, towards both the North Pole and South Pole, there were no daytime acquisitions for Landsat-8. The mean average latitudinal values start to decrease above the 75.6° N in the North Pole and 80.0° S in the South Pole for both Sentinel-2A and -2B and reach 13.34 (Sentinel-2A) and 9.82 (Sentinel-2B) at 82.8° N and 5.95 (Sentinel-2A) and 13.10 (Sentinel-2B) at 83.6° S. There were no daytime acquisitions for Sentinel-2A -2B above 82.8° N and 83.6° S, towards both the North Pole and South Pole.

### 4.3. Global Cloud-Weighted Number of Observations for Landsat-8 and Sentinel-2A -2B

**Figure 6:**
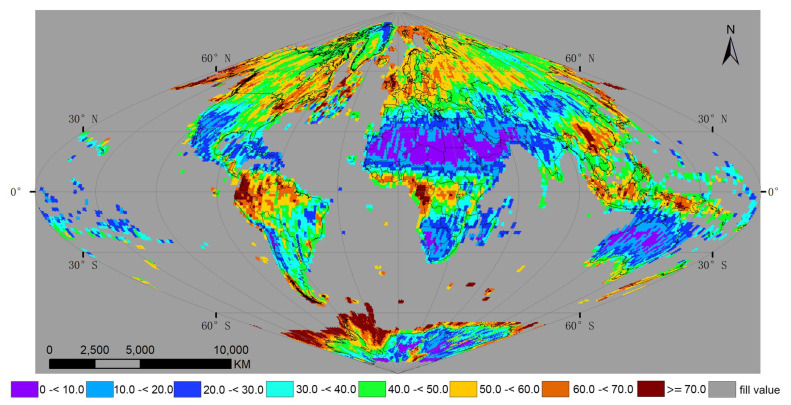
Average percent cloud cover of Landsat-8 satellite observations over each land grid point in 2018. The results were examined on a global grid defined by an equal area sinusoidal projection, composed of 7201 × 3601 grid points, with a spacing of 0.05°.

Figure 6 shows the global average percent cloud cover examined over land grid points that had at least one Landsat-8 daytime observation in 2018. Typically, high cloud cover occurred over tropical rainforest areas near the equator, while desert and dryland areas typically had low cloud cover. The global mean average percent cloud cover derived from all Landsat-8 daytime observations for 2018 defined on the global equal area sinusoidal projection was 0.41.

**Figure 7:**
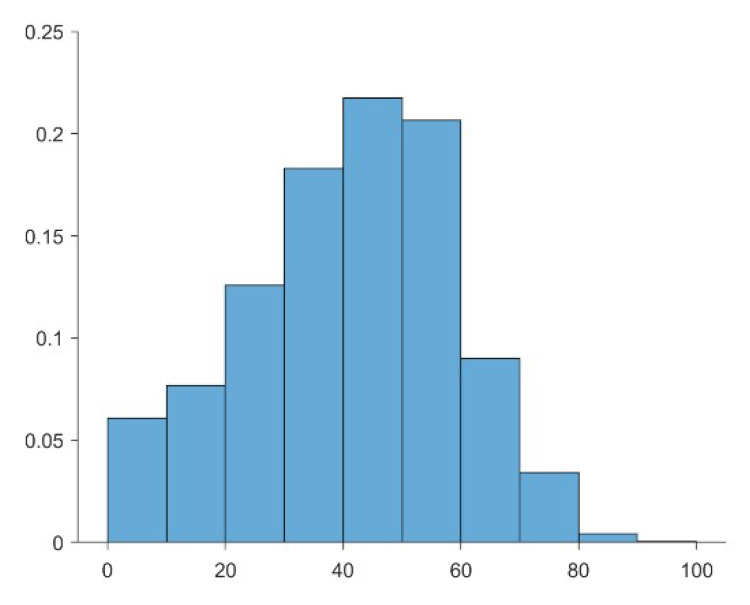
Histogram of global average percent cloud cover for Landsat-8 observations. The bin widths of histogram are defined at 10% intervals, and the percentages of each bin denote the number of observations compared with the total number. The data are from Figure 6.

Figure 7 shows histograms of the global average percent cloud cover data (Figure 6). The data for Landsat-8 average percent cloud cover were asymmetrically distributed, with a lower limit of 0 cutting the curve. Across the global map of average percent cloud cover data, the most common values were 0.4 to 0.5 for Landsat-8, occurring at 20.75% of the global grid points.

**Figure 8:**
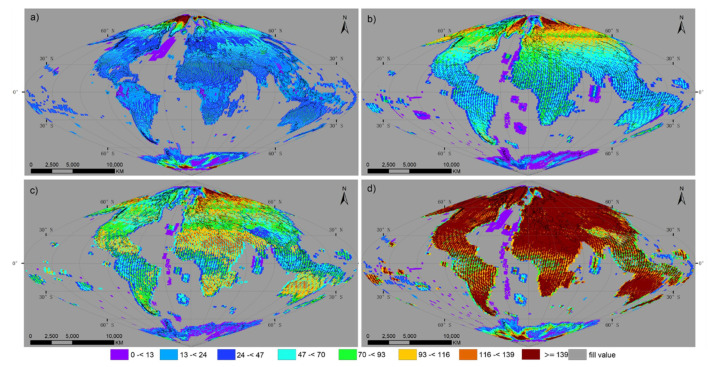
Cloud-weighted total number of satellite observations over each land grid point from 1 January to 31 December 2018 by (**a**) Landsat-8, (**b**) Sentinel-2A, (**c**) Sentinel-2B, and (**d**) three sensors combined. The results were examined on a global grid, defined by an equal area sinusoidal projection, composed of 7201 × 3601 grid points, with a spacing of 0.05°.

Figure 8 shows the cloud-weighted number of observations for each global grid point for (a) Landsat-8, (b) Sentinel-2A, (c) Sentinel-2B, and (d) three sensors combined in 2018. The geographical distribution pattern of cloud-weighted observations is complex and irregular. Generally, data availability was influenced by the sensor combination, data reception strategy and the system mission constraints (Figure 3). Meanwhile, more observations were carried out at high latitudes, related to greater lateral orbit swath overlap in those areas. In addition to these factors, the cloud-weighted number of observations determined the cloud contamination level of all data. Thus, areas with frequent high cloud cover, e.g., tropical rainforests, were more severely contaminated by clouds, while low cloud cover over deserts and drylands ensured a higher probability of clear view observations. Overall, the global mean average cloud-weighted number of observations for (a) Landsat-8, (b) Sentinel-2A, (c) Sentinel-2B, (d) and three sensors in 2018 was 20.37, 33.67, 36.43, and 81.86, respectively.

### 4.4. Comparison of Cloud-Weighted Number of Observations over Three Selected Locations for the Year 2018

Table 2 summarises the number of observations, cloud-weighted observations, and clear views, as well as accuracy levels, for the three selected locations for the year 2018. The accuracy is 98.7%, 91.0% and 81.7% for Algeria, (30.0° N, 0.0°), Brazil (3.138° S, 62.180° W) and Sweden (56.842° N, 15.057° E), respectively, evaluated by comparing the number of cloud-weighted observations (42.45, 9.81, 18.93) with the number of clear views (43, 9, 16). Table 3 shows the acquisition date, path, row and cloud condition of Landsat-8 acquisition imageries covering the selected location in the northwest of Algeria (30.0° N, 0.0°) in the year 2018. The total number of observations covering Algeria (30.0° N, 0.0°) in the year 2018 is 46, which is consistent with the value dumping from Figure 2. All through the year 2018, the observation condition was excellent for Landsat-8 image acquisition in the northwest of Algeria, Sahara desert (30.0° N, 0.0°), with 43 clear views, except the observations acquired on 8 January 2018, 2 February 2018 and 7 October 2018, which were contaminated by high confidence cloud.

## 5. Discussion

The data availability of satellite observations influences surface land monitoring capabilities. Having more observations in a given time enables more reliable time series fitting [33,34], higher precision land cover classification [35], improved stable land change detection [36] and more cloud-free composited products [37,38]. The global spatial coverage of satellite observations enables the large area monitoring, i.e., on a regional or global scale, of land cover change [39] and the mapping of burned areas [40]. The polar-orbiting Landsat-8 satellite has even acquired high latitude area observations that enable ice flow mapping [23].

The combination of the Landsat-8 -9 and Sentinel-2A -2B four sensors together was enabled to develop a dense time series, improving the ability to detect abrupt land cover changes, and monitor phenology variations at a specific time period [41,42]. Combining the four sensors could offer a higher temporal resolution, addressing the gap of the observation sample data for model training, caused by the cloud obscuration and data missing issue by system [43].

More sensors are combined to facilitate more observations, shorter revisit interval between consecutive observations will be got. With similar multi-spectral bands, Sentinel-2A -2B and Landsat-8 -9 combined together provide 10–30-m resolution global land coverage. Compared to the 2.9 days from the combinations of Landsat-8 and Sentinel-2A -2B, the global median average revisit interval for the four sensors combined are 2.3 days. This increase in revisit interval was not striking, as only one Landsat was added in and also because Sentinel-2 has a wider swath coverage than Landsat. The combination of the four moderate-resolution sensors could still advance the solution for near daily temporal coverage that can benefit for many applications, etc., drought monitoring [44] and evapotranspiration estimations [45].

The global mean average number of observations was 162.6 for the combination of Landsat-8 and Sentinel-2A -2B in 2018, derived from the orbit swath model [4]. In this study, the global mean average number of observations derived from daytime surface observations of three sensors combined was 141.40, reduced by 13.0%. The global mean average number of observations derived from the orbit swath model (162.6) considers orbit swath geometry and assumes that at each location an observation is acquired with equal opportunity, without considering data acquisition strategy, system reception ability or instrument issues. Thus, the 13.0% reduction gives a global overall estimation of the influence of date acquisition strategy and instrument issues on data availability. Effects of cloud cover on satellite images used for surface monitoring are important. The cloud-weighted number of observations, compared with surface land number of observations, taking into account cloud obscuration, reduced the global average number of observations of three sensors combined from 141.4 to 81.9, i.e., by 42.1%.

Three different locations: Algeria, (30.0° N, 0.0°), Brazil (3.138° S, 62.180° W) and Sweden (56.842° N, 15.057° E) were used to evaluation the accuracy of the cloud-weighted number of observations by comparing with the clear views from the Landsat-8 surface reflectance observations. The accuracy achieved 98.7%, 91.0% and 81.7% for three locations, respectively. The results show an appropriate accuracy for using the cloud-weighted number of observations to estimate the useful clear view observations considering cloud contamination.

Orbit swath geometry, with increased lateral orbit swath overlap at higher latitudes, has a significant effect on data availability. Latitudinal average number of observations increases from the equator at 0° (25.4) towards north to 81.2° N (295.7) for Landsat-8, from 0° (50.3) to 75.6° N (117.0) for Sentinel-2A and from 0° (51.5) to 67.6° N (109.2) for Sentinel-2B, respectively. As all of the three sensors acquire observations with different probabilities at different latitudes according to their data reception strategy [8], this is not a single factor analysis. Solar geometry, i.e., the annual progression of the solar position, denotes the geographical latitudinal coverage of observations that can be acquired during the daytime. The maximum latitudinal coverage towards the south for Landsat-8 is 55.08° S in the month of June (summer solstice), and towards the north is 66.71° N in the month of December (winter solstice).

In this study, the surface observation availability was examined on a tile level. The cloud-weighted number of observations assumed that each pixel showing cloud in the image was the same and equal to the percentage of the image being cloudy. Consequently, the shape and exact location of clouds over the area was not clear. However, the reported results give an overall evaluation of cloud-free observation areas in each image frame. The complicated pattern of data availability related to cloud was apparent in the global-scale map.

## 6. Conclusions

This study demonstrates that sensor combination, system reception, orbit geometry, solar geometry, and cloud contamination could all influence data availability. The main findings of the research were as follows: (i) Sensor combination enabled more observations and shorter revisit intervals between consecutive observations. The global median average revisit intervals for various combinations were: 8.0 d for Landsat-8 and Landsat-9, 3.7 d for Sentinel-2A and Sentinel-2B, and only 2.3 d when all four sensors were combined; (ii) The global mean average number of surface observations for the combination of Landsat-8 and Sentinel-2A -2B is 141.4; (iii) The global mean average cloud-weighted number of observations is 81.9 for the three sensors combined; (iv) Landsat-8 surface reflectance covering three different locations was used to compare the cloud-weighted number of observations. The results show an overall accuracy of more than 80%.

Given its similar spectral and spatial characteristics as Landsat-8/9, Sentinel-2A/2B data could be combined with Landsat data to provide better moderate-resolution imaging. The modeled orbit swath data obtained from COVE was used to analyse the revisit interval between consecutive observations from combined sensors. Future work could include Sentinel-2 surface land observations combined with Landsat ones to derive the surface land observation analysis at global scales for the combination of Landsat-8/9 and Sentinel-2A/2B.

## Figures and Tables

**Table 1:** Global statistics including mean, median and various modes for combinations of (a) Landsat-8 and Landsat-9, (b) Sentinel-2A and Sentinel-2B, and (c) Landsat-8, Landsat-9, Sentinel-2A and Sentinel-2B from 1 January to 31 December 2016. Results are given to three decimal places. Percentages for each mode are tabulated. All data are from Figure 1.

	Landsat-8 Landsat-9	Sentinel-2A Sentinel-2B	Landsat-8 Landsat-9 Sentinel-2A Sentinel-2B
Mean Total	6.042	3.795	2.277
Median Total	7.999	3.667	2.342
Most Frequent Total Value	8.000 (31.7%)	5.000 (29.0%)	3.076 (7.3%)
2nd Most Frequent	3.967	3.333	2.342
Total Value	(13.3%)	(13.6%)	(4.2%)
3rd Most Frequent	3.999	2.486	1.525
Total Value	(2.8%)	(0.2%)	(1.8%)

**Table 2:** Number of observations, number of cloud-weighted observations, number of clear views and the accuracy of Landsat-8 acquisition imageries covering the three selected locations in the year 2018.

Location	Number of Observations	Number of Cloud-Weighted Observations	Number of Clear Views	Accuracy
Algeria (30.0° N, 0.0°)	46	42.45	43	98.7%
Brazil (3.138° S, 62.180° W)	23	9.81	9	91.0%
Sweden (56.842° N, 15.057° E)	44	18.93	16	81.7%

**Table 3:** Acquisition date, path, row, cloud condition of Landsat-8 acquisition imageries covering the selected location in the northwest of Algeria (30.0° N, 0.0°) in the year 2018.

Acquisition Date	Path	Row	Cloud Condition	Acquisition Date	Path	Row	Cloud Condition
2018-01-01	196	39	Clear	2018-07-03	197	39	Clear
2018-01-08	197	39	High confidence	2018-07-12	196	39	Clear
2018-01-17	196	39	Clear	2018-07-19	197	39	Clear
2018-01-24	197	39	Clear	2018-07-28	196	39	Clear
2018-02-02	196	39	High confidence	2018-08-04	197	39	Clear
2018-02-09	197	39	Clear	2018-08-13	196	39	Clear
2018-02-18	196	39	Clear	2018-08-20	197	39	Clear
2018-02-25	197	39	Clear	2018-08-29	196	39	Clear
2018-03-06	196	39	Clear	2018-09-05	197	39	Clear
2018-03-13	197	39	Clear	2018-09-14	196	39	Clear
2018-03-22	196	39	Clear	2018-09-21	197	39	Clear
2018-03-29	197	39	Clear	2018-09-30	196	39	Clear
2018-04-07	196	39	Clear	2018-10-07	197	39	High confidence
2018-04-14	197	39	Clear	2018-10-16	196	39	Clear
2018-04-23	196	39	Clear	2018-10-23	197	39	Clear
2018-04-30	197	39	Clear	2018-11-01	196	39	Clear
2018-05-09	196	39	Clear	2018-11-08	197	39	Clear
2018-05-16	197	39	Clear	2018-11-17	196	39	Clear
2018-05-25	196	39	Clear	2018-11-24	197	39	Clear
2018-06-01	197	39	Clear	2018-12-03	196	39	Clear
2018-06-10	196	39	Clear	2018-12-10	197	39	Clear
2018-06-17	197	39	Clear	2018-12-19	196	39	Clear
2018-06-26	196	39	Clear	2018-12-26	197	39	Clear
